# Comparison of repeatability of swept-source and spectral-domain optical coherence tomography for measuring inner retinal thickness in retinal disease

**DOI:** 10.1371/journal.pone.0210729

**Published:** 2019-01-16

**Authors:** Eun Hee Hong, So Jung Ryu, Min Ho Kang, Mincheol Seong, Heeyoon Cho, Jong Hun Yeom, Yong Un Shin

**Affiliations:** 1 Department of Ophthalmology, Hanyang University College of Medicine, Seoul, Korea; 2 Department of Anesthesiology and Pain Medicine, Hanyang University College of Medicine, Seoul, Korea; Boston Medical Center, Boston University School of Medicine, UNITED STATES

## Abstract

**Purpose:**

To compare repeatability between SS-OCT and SD-OCT for measurement of macular, macular retinal nerve fiber (mRNFL), and ganglion cell-inner plexiform layer (GC-IPL) thickness in various retinal diseases.

**Methods:**

One hundred and fourteen eyes of 114 subjects were investigated. Seventy-eight eyes with retinal disease and 36 normal eyes underwent two consecutive measurements of macular, mRNFL, and GC-IPL thickness using SS-OCT and SD-OCT. The data were obtained using the Early Treatment Diabetic Retinopathy Study (ETDRS) protocol. The eyes with retinal diseases were divided into three subgroups according to central macular thickness (CMT) for analysis. The intraclass correlation coefficient (ICC) was calculated to determine the repeatability of OCT device.

**Results:**

In normal eyes, both OCT devices showed excellent repeatability of macula, mRNFL, and GC-IPL thickness measurements with high ICCs in all ETDRS subfields. In eyes with retinal disease, although SS-OCT showed better repeatability for inner retinal thickness measurements than SD-OCT, the overall ICCs were lower than those in normal eyes. In subgroup analysis, the ICCs in the low CMT group were lower than those in the normal and high CMT groups, particularly when using SD-OCT.

**Conclusions:**

Both OCT devices had comparable repeatability for retinal thickness measurement in normal eyes and eyes with retinal disease. However, the possibility of measurement error should be considered in eyes with a thin and atrophic retina.

## Introduction

Optical coherence tomography (OCT) has been developed over recent decades and can quantify the thickness of the retinal layer noninvasively.[1] OCT has been considered an essential device in both clinical and research fields because it provides quantitative data as well as qualitative information when assessing retinal status. However, several factors, including media opacity, machine resolution, capture speed, auto-segmentation software, and patient cooperation, can affect the repeatability of OCT measurements, and may cause serious errors in evaluation of a patient’s condition or interpretation of research data. Therefore, whenever a new OCT device is developed, a repeatability assessment is needed to ensure its reliability.[2–4]

OCT devices and software algorithms for auto-segmentation have evolved to the extent that they can now segment the microstructure of the retina and provide quantitative thickness data for specific region, including the inner retina [5, 6] and choroid [7, 8] as well as the macula. Measurement of the thickness of the inner retina is important in the field of glaucoma research, especially in early diagnosis and progression analysis; therefore, investigators have evaluated the repeatability of various types of OCT devices and have reported high repeatability for measurements of the thickness of the peripapillary retinal nerve fiber layer (RNFL) and the thickness of the inner retina in the diagnosis of glaucoma.[9, 10] Assessment of the inner retinal thickness is also important in macular diseases, such as epiretinal membrane, macular hole, and retinal vein occlusion, as the RNFL and ganglion cell-inner plexiform layer (GC-IPL) thickness are thought to be important in visual prognosis.[11–13]

Recently, swept-source OCT (SS-OCT) has been introduced commercially. SS-OCT provides longer wavelength light sources than spectral domain-OCT (SD-OCT),[14] and has a more rapid scanning speed and provides better images of the deep retinal structures when compared with SD-OCT.[15–17] A report by Mansouri et el demonstrated high repeatability using SS-OCT for measurement of the inner retinal, macula, and choroidal layers in normal eyes.[18] Lee et al. reported high repeatability of both SD-OCT and SS-OCT for measurement of the peripapillary RNFL and GC-IPL in normal subjects.[19] In retina diseases, the auto-segmentation may be affected, which may impair the reliability of the measurements in both devices. Other studies have reported the repeatability of measurements of inner retinal thickness in retinal diseases using SD-OCT.[20, 21] However, no study has compared repeatability between SD-OCT and SS-OCT in eyes with retinal disease. This study investigated the intra-device repeatability of thickness measurements for the macula and GC-IPL layers in the macular area in normal eyes and eyes with various retinal diseases using SS-OCT and SD-OCT and compared the repeatability of the two devices.

## Materials and methods

### Participants

This cross-sectional study design was approved by the Institutional Review Board (IRB) of Hanyang University Guri Hospital, Gyunggi-do, Korea, and all research conducted adhered to the tenets of the Declaration of Helsinki (IRB no. 2017-10-013). Written informed consent was obtained from all participants, which was approved by the IRB. One hundred and twenty-seven subjects from the Department of Ophthalmology of Hanyang University Guri Hospital were enrolled in the study between October 2017 and January 2018. The subjects underwent a complete ophthalmic examination, including best-corrected visual acuity (BCVA), intraocular pressure (Goldman applanation tonometry), refractive errors, slit lamp biomicroscopy, fundoscopy, and OCT, along with a complete medical history. Only one eye per subject was included. In the normal group, the right eye was selected. In the disease group, if both eyes were affected, the right eye was selected, otherwise the affected eye was selected.

The retinal disease group (83 eyes, 83 patients) included 25 eyes with age-related macular degeneration (AMD), 12 with retinal vein occlusion (RVO), 31 with diabetic retinopathy (DR), 5 with central serous chorioretinopathy (CSC), 7 with epiretinal membrane (ERM), and 3 with ocular Behcet’s disease. Subjects with a BCVA of less than 20/200 were excluded to avoid fixation failure.

The normal group (36 eyes, 36 healthy subjects) was age-matched and included subjects at least 19 years with a BCVA of 20/25 or higher, a normal fundus, and an intraocular pressure <21 mmHg. Participants in both groups were excluded if they had a spherical equivalent greater than ± 6 D, cataract graded as more severe than grade 3 (Lens Opacities Classification System III),[22] a history of glaucoma or optic nerve disease, or a history of vitrectomy.

### Optical coherence tomography imaging

All subjects underwent imaging with an SS-OCT (deep range imaging OCT, Triton, Topcon, Tokyo, Japan) and SD-OCT (3D-OCT-2000, Topcon) in the same clinical setting. The two OCT examinations were performed twice by one experienced technician in the order of SD-OCT and SS-OCT, respectively. There was a 10-minute interval between examinations using the two OCT devices. The pupil was dilated before examination and an internal fixation target in the OCT device was used. Unacceptable images, such as those with poor scan quality (an image quality score provided by the onboard OCT software lower than 45, according to manufacturer’s recommendation [23, 24]) obtained by either OCT device, were excluded. The images were interpreted using the same image viewer (IMAGEnet 6 Version 1.20.11109, Topcon) for both OCT devices. A three-dimensional vertical scan protocol composed of 512 × 128 scans (512 A-scans for each of 128 B-scans, for a total of 65,536 axial scans/volume) was used for both OCT devices. The scan length was 7.0 × 7.0 mm for both SS-OCT and SD-OCT. SD-OCT uses a super luminescence diode at 840 nm wavelength as light source, and provides 5–6 μm of axial resolution, 20 μm of transverse resolution and maximum scan velocity of 27,000 A-scans per second. SS-OCT uses a tunable laser as a light source to provide a 1050 nm centered wavelength and provides 8 μm of axial resolution, 20 μm of transverse resolution and maximum scan velocity of 100,000 A-scans per second.[25] The macular, macular RNFL (mRNFL), and GC-IPL thicknesses were measured automatically.[26] All measurements were presented in the Early Treatment Diabetic Retinopathy Study (ETDRS) protocol with the central circle, inner ring, and outer ring using 1-, 3-, and 6-mm diameters. Manual correction of segmentation was not applied.

### Statistical analysis

All statistical analyses were performed using SPSS version 22.0 (IBM Corp., Armonk, NY USA). Data are presented as mean values ± standard deviation. The repeatability of the two consecutive measurements was assessed using the intraclass correlation coefficient (ICC) and the coefficient of variation (CV). The ICC was used to determine the repeatability for each device using a one-way random-effects model. The degree of reliability was classified according to the ICC as follows: slight (0–0.2), fair (0.21–0.4), moderate (0.41–0.6), substantial (0.61–0.8), and almost perfect (0.81–1).[27] To compare the 2 ICC values for intradevice repeatability between the 2 types of OCT systems, we used the statistical method of Donner and Zou.[28] A CV of <10% indicates high repeatability. Bland-Altman plots with regression analysis were used to analyze the agreement between the thickness values obtained by each OCT device. Statistical significance was defined as *P* < 0.05. We divided the retinal disease group into 3 subgroups for analysis according to central macular thickness (CMT), i.e., low CMT (<200 mm), normal CMT (200–300 mm), and high CMT (>300 mm) based on a previous study.[20]

## Results

### Demographics

One hundred and fourteen eyes (114 patients) met the study inclusion criteria. Five eyes were excluded because of poor image quality (n = 2) or decentration (n = 3). There were no significant differences in age, sex, laterality, refractive errors, or intraocular pressure between the normal and retinal disease groups. However, the retinal diseases group showed significantly lower BCVA than the normal group (*p* = 0.009). There was no significant difference in clinical features in the retinal disease group, except for a significantly lower BCVA in the low CMT group (*p* = 0.012; Table 1). The low CMT group included 20 eyes with diagnoses such as geographic atrophy in AMD, macular atrophy associated with RVO, macular atrophy associated with DR, and ocular Behcet’s disease. The normal CMT group included 35 eyes with AMD, RVO, DR, CSC, and ocular Behcet’s disease. The high CMT group included 23 eyes with AMD, macular edema secondary to RVO, diabetic macular edema, CSC, and ERM with macular edema. The demographics and clinical characteristics of the study subjects are shown in Table 1.

**Table 1:** Demographics and clinical characteristics of study patients.

	Normal group (n = 36)	Retinal disease group (n = 78)					
	Total	Total	*P-*value†	Low CMT (n = 20)	Normal CMT (n = 35)	High CMT (n = 23)	*P-*value‡
**Age, years**	56.1 ± 15.8	60.9 ± 13.9	0.343	58.3 ± 11.1	62.1 ± 14.3	60.3 ± 12.8	0.546
**Sex, male:female**	22:14	38:40	0.398	12:8	18:17	8:15	0.346
**Laterality, OD:OS**	18:18	43:35	0.234	8:12	20:15	15:8	0.432
**BCVA, logMAR**	0.04 ± 0.09	0.58 ± 1.01	0.009	1.94 ± 1.90	0.33 ± 0.58	0.39 ± 0.36	0.012
**Refractive error, (SE, diopters)**	-0.54 ± 1.76	-0.63 ±2.54	0.061	-1.15 ± 1.50	-0.34 ± 2.82	-0.85 ± 2.50	0.058
**Intraocular pressure**	16.01 ± 3.32	16.41 ± 3.58	0.483	16.40 ±3.13	16.86 ± 2.85	15.70 ± 4.66	0.593
**Diagnosed diseases**							
AMD		22		3	14	5	
RVO		12		5	3	4	
DR		29		10	16	3	
CSC		5		0	1	4	
ERM		7		0	0	7	
Ocular Behcet’s disease		3		2	1	0	

^†^Independent t-test.

^‡^One way analysis of variance

*P* < 0.05 indicates statistically significant difference

AMD = age-related macular degeneration; BCVA = best-corrected visual acuity; CMT = central macular thickness; CSC = central serous chorioretinopathy; DR = diabetic retinopathy; ERM = epiretinal membrane; IOP = intraocular pressure; RVO = retinal vein occlusion; SE = spherical equivalent

### Repeatability in normal eyes

**Fig 1:**
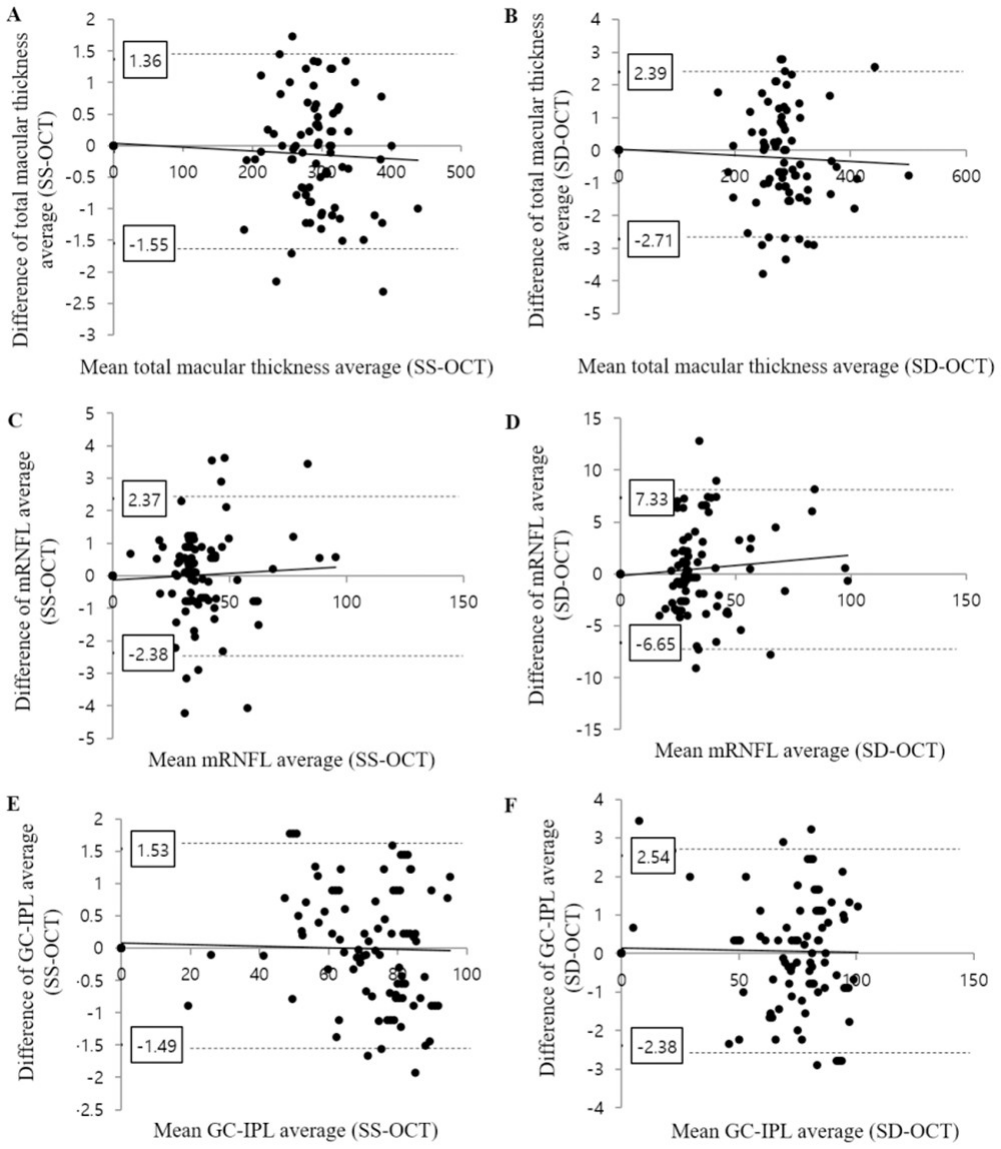
Bland–Altman plot analyses of the difference between two consecutive measurements of swept source optical coherence tomography (SS-OCT) and spectral domain optical coherence tomography (SD-OCT). (A-B) Total macular thickness measurements by SS-OCT (A) and SD-OCT (B). (C-D) Macular retinal nerve fiber layer (mRNFL) thickness measurements by SS-OCT (C) and SD-OCT (D). (E-F) Macular ganglion cell-inner plexiform layer (GC-IPL) thickness measurements by SS-OCT (E) and SD-OCT (F).

In the normal eyes, the ICCs for the macula, mRNFL, and GC-IPL thickness measurements exceeded 0.9 in most subfields and were classified as almost perfect (ICC = 0.81–1) for SS-OCT and SD-OCT. The CV values were generally high. Only the central thickness (0.817) and outer temporal subfield (0.840) measurements of the mRNFL using SD-OCT were under 0.9. The ICC was 0.993 for SS-OCT and 0.990 for SD-OCT for the average macula thickness (*p* = 0.136), 0.989 for SS-OCT and 0.969 for SD-OCT, respectively, for the average mRNFL thickness (*p* < 0.001), and 0.991 for SS-OCT and 0.989 for SD-OCT, respectively, for the average GC-IPL thickness (*p* = 0.269) (Fig 1, Table 2).

**Table 2:** Intra-device repeatability of the two consecutive measurements using the intraclass correlation coefficient and coefficient of variation in normal eyes and eyes with retinal disease.

			Normal group					Retinal disease group				
			SS-OCT		SD-OCT			SS-OCT		SD-OCT		
			ICC (95% CI)	CV (%)	ICC (95% CI)	CV (%)	*P*†	ICC (95% CI)	CV (%)	ICC (95% CI)	CV (%)	*P*†
**Macula**		**Center**	0.983 (0.966–0.991)	1.0	0.975 (0.900–0.994)	1.6	0.116	0.971 (0.952–0.982)	2.5	0.931 (0.888–0.957)	5.5	0.036
	**Inner**	**Temporal**	0.995 (0.990–0.997)	0.5	0.990 (0.961–0.998)	1.0	0.017	0.984 (0.974–0.990)	2.1	0.976 (0.961–0.985)	2.9	0.203
		**Superior**	0.996 (0.992–0.998)	0.4	0.989 (0.958–0.997)	1.0	0.001	0.966 (0.944–0.979)	2.9	0.954 (0.925–0.971)	5.2	0.266
		**Nasal**	0.995 (0.991–0.998)	0.5	0.982 (0.928–0.996)	1.4	<0.001	0.949 (0.918–0.969)	3.5	0.968 (0.949–0.981)	5.1	0.343
		**Inferior**	0.993 (0.986–0.996)	0.6	0.993 (0.974–0.998)	0.8	0.5	0.967 (0.947–0.980)	3.0	0.987 (0.980–0.992)	2.0	0.028
	**Outer**	**Temporal**	0.995 (0.932–0.982)	0.5	0.989 (0.957–0.997)	1.1	0.008	0.977 (0.963–0.986)	2.8	0.969 (0.949–0.981)	3.7	0.269
		**Superior**	0.988 (0.977–0.994)	0.8	0.987 (0.978–0.999)	1.2	0.402	0.958 (0.932–0.974)	3.0	0.956 (0.929–0.973)	4.0	0.462
		**Nasal**	0.982 (0.965–0.991)	0.9	0.987 (0.978–0.999)	1.2	0.158	0.932 (0.889–0.958)	6.7	0.962 (0.938–0.976)	5.4	0.113
		**Inferior**	0.991 (0.983–0.996)	0.8	0.993 (0.970–0.998)	0.9	0.220	0.977 (0.963–0.986)	3.3	0.974 (0.958–0.984)	3.5	0.401
		**Average**	0.993 (0.990–0.995)	0.7	0.990 (0.985–0.996)	1.1	0.136	0.989 (0.983–0.993)	3.3	0.979 (0.967–0.987)	4.1	0.093
**mRNFL**		**Center**	0.950 (0.903–0.975)	3.7	0.817 (0.642–0.907)	11.7	<0.001	0.940 (0.903–0.963)	4.6	0.823 (0.727–0.981)	10.9	0.010
	**Inner**	**Temporal**	0.957 (0.917–0.978)	3.8	0.919 (0.842–0.959)	8.9	0.023	0.942 (0.906–0.964)	5.6	0.934 (0.869–0.967)	5.0	0.393
		**Superior**	0.982 (0.965–0.991)	1.2	0.985 (0.971–0.993)	1.3	0.287	0.771 (0.651–0.821)	20.4	0.739 (0.581–0.819)	25.6	0.381
		**Nasal**	0.968 (0.936–0.983)	2.8	0.902 (0.711–0.982)	9.1	<0.001	0.867 (0.784–0.918)	9.9	0.855 (0.714–0.927)	10.3	0.425
		**Inferior**	0.976 (0.952–0.988)	2.7	0.973 (0.947–0.986)	3.0	0.357	0.899 (0.836–0.938)	9.0	0.881 (0.764–0.940)	9.8	0.362
	**Outer**	**Temporal**	0.918 (0.839–0.958)	4.2	0.840 (0.686–0.918)	10.5	0.015	0.858 (0.769–0.912)	10.1	0.784 (0.572–0.891)	20.5	0.175
		**Superior**	0.982 (0.965–0.991)	1.0	0.985 (0.970–0.992)	1.2	0.287	0.948 (0.915–0.968)	5.0	0.870 (0.841–0.885)	9.8	0.026
		**Nasal**	0.987 (0.974–0.993)	1.0	0.944 (0.890–0.972)	4.4	<0.001	0.882 (0.809–0.927)	10.2	0.848 (0.699–0.923)	11.1	0.291
		**Inferior**	0.991 (0.982–0.995)	0.9	0.946 (0.893–0.973)	4.5	<0.001	0.955 (0.926–0.972)	6.1	0.936 (0.875–0.967)	5.1	0.231
		**Average**	0.989 (0.979–0.995)	2.4	0.969 (0.940–0.984)	6.1	<0.001	0.932 (0.889–0.958)	9.0	0.913 (0.857–0.959)	12.0	0.301
**GC-IPL**		**Center**	0.975 (0.900–0.994)	1.5	0.931 (0.888–0.957)	4.5	<0.001	0.953 (0.906–0.991)	4.0	0.941 (0.852–0.982)	5.7	0.318
	**Inner**	**Temporal**	0.990 (0.961–0.998)	0.9	0.976 (0.961–0.985)	3.4	0.003	0.985 (0.990–0.997)	2.5	0.974 (0.944–0.990)	3.4	0.130
		**Superior**	0.989 (0.958–0.997)	1.0	0.954 (0.925–0.971)	4.0	<0.001	0.986 (0.982–0.998)	2.6	0.946 (0.914–0.979)	4.6	0.003
		**Nasal**	0.982 (0.928–0.996)	1.2	0.968 (0.949–0.981)	4.1	0.037	0.975 (0.951–0.998)	2.9	0.949 (0.918–0.969)	4.8	0.070
		**Inferior**	0.993 (0.974–0.998)	0.8	0.987 (0.980–0.992)	1.0	0.028	0.953 (0.926–0.986)	4.6	0.967 (0.947–0.980)	4.0	0.232
	**Outer**	**Temporal**	0.989 (0.957–0.997)	1.2	0.969 (0.949–0.981)	2.2	0.001	0.965 (0.932–0.982)	2.3	0.967 (0.943–0.986)	4.1	0.452
		**Superior**	0.997 (0.988–0.999)	0.5	0.956 (0.929–0.973)	3.9	<0.001	0.988 (0.977–0.994)	1.9	0.951 (0.922–0.974)	4.4	0.002
		**Nasal**	0.997 (0.988–0.999)	0.5	0.962 (0.938–0.976)	2.4	<0.001	0.982 (0.965–0.991)	2.2	0.932 (0.889–0.958)	6.0	0.003
		**Inferior**	0.993 (0.970–0.998)	0.7	0.974 (0.958–0.984)	2.1	<0.001	0.971 (0.953–0.996)	3.0	0.967 (0.933–0.986)	4.1	0.395
		**Average**	0.991 (0.987–0.994)	0.9	0.989 (0.983–0.993)	3.1	0.269	0.981 (0.971–0.994)	2.9	0.979 (0.967–0.987)	4.6	0.419

^†^Statistical method of Donner and Zou.

*P* < 0.05 indicates statistically significant difference; A CV of <10% indicates high repeatability

ICC = intraclass correlation coefficient; CI = confidence interval; CV = coefficient of variation; SS = swept-source optical coherence tomography; SD = spectral domain optical coherence tomography; mRNFL = macular retinal nerve fiber layer; GC-IPL = ganglion cell-inner plexiform layer

### Repeatability in eyes with retinal disease

In the eyes with retinal disease, the ICCs for the average thickness measurements were lower for both OCT measurements than those in the normal group (normal vs retinal disease group: 0.993 vs. 0.989 for the macula, 0.989 vs. 0.932 for the mRNFL, and 0.991 vs. 0.981 for the GC-IPL when using SS-OCT; 0.990 vs. 0.979 for the macula, 0.969 vs. 0.913 for the mRNFL, and 0.989 vs. 0.979 for the GC-IPL using SD-OCT) (Fig 1). In each subfield, most ICCs in the eyes with retinal disease were lower than those in the normal group for both OCT devices. There were the exceptions, i.e., the central thickness of the mRNFL and GC-IPL using SD-OCT (normal group vs. retinal disease group: 0.817 vs. 0.823 for the mRNFL and 0.931 vs. 0.941 for the GC-IPL). The overall ICCs for the average thickness measurements for SS-OCT were higher than those for SD-OCT, and the CV results were similar. In terms of the repeatability of measurement for the mRNFL, the inner superior subfield and the outer temporal subfield of the quadrant map showed ICCs lower than 0.8 (inner superior, 0.771 on SS-OCT and 0.739 on SD-OCT; outer temporal, 0.858 on SS-OCT and 0.784 in SD-OCT). No subfield had an ICC lower than 0.8 for the macular or GC-IPL thickness measurement (Table 2).

**Fig 2:**
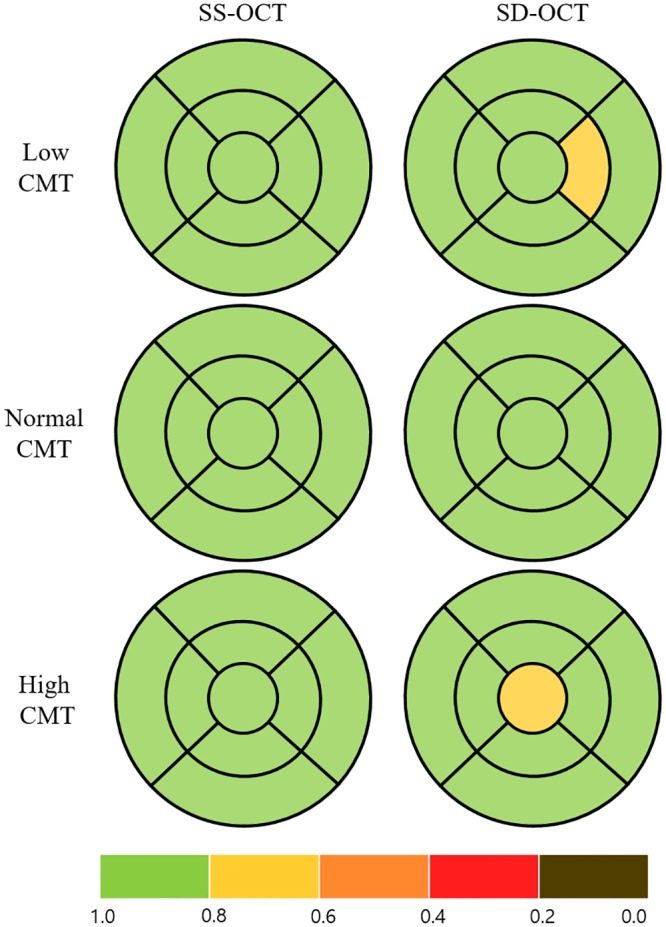
Early Treatment Diabetic Retinopathy Study subfield maps with color scales according to intraclass correlation coefficient (ICC) values to represent the repeatability of two consecutive measurements of macular thickness in eyes with retinal disease. The maps were drawn by assuming the right eye. The color scale bar at the bottom represents ICC values. CMT = central macular thickness.

**Fig 3:**
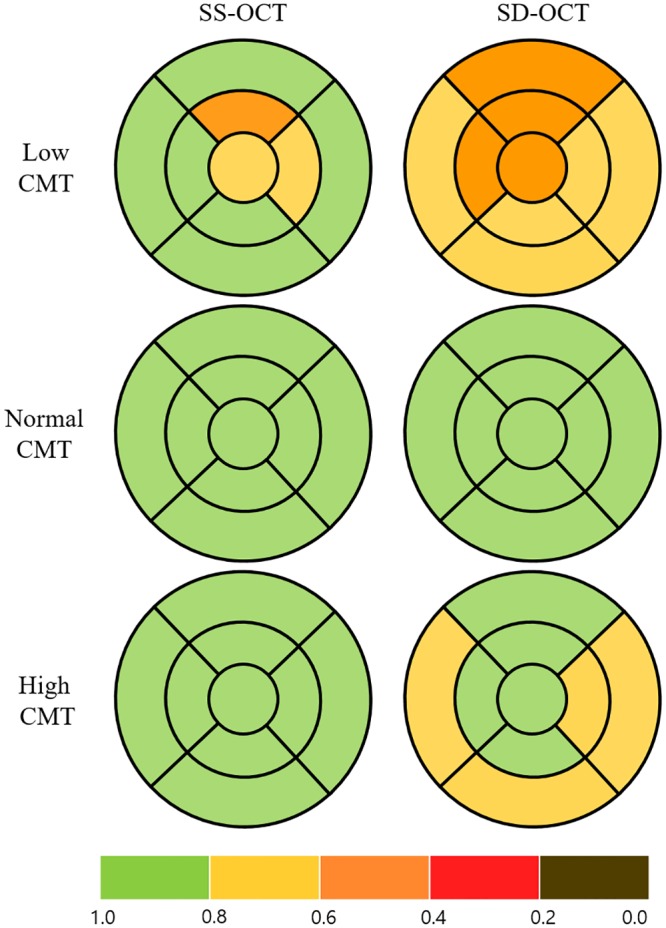
Early Treatment Diabetic Retinopathy Study subfield maps with color scales according to intraclass correlation coefficient values to represent the repeatability of the consecutive measurements of macular retinal nerve fiber layer thickness in eyes with retinal disease. The maps were drawn by assuming the right eye. The color scale bar at the bottom represents ICC values. CMT = central macular thickness.

**Fig 4:**
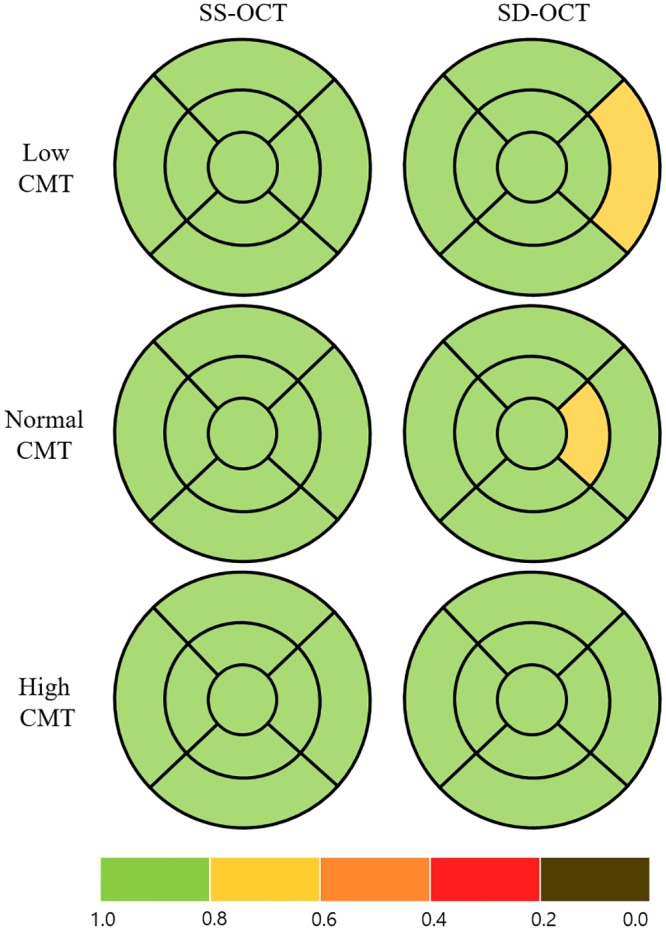
Early Treatment Diabetic Retinopathy Study subfield maps with color scales according to intraclass correlation coefficient (ICC) values to represent the repeatability of two consecutive measurements of macular ganglion cell-inner plexiform layer thickness in eyes with retinal disease. The maps were drawn by assuming the right eye. The color scale bar at the bottom represents ICC values. CMT = central macular thickness.

We compared the repeatability of thickness measurements for the macula, mRNFL, and GC-IPL in three subgroups according to CMT (low, normal and high). The ICCs for macular thickness showed high repeatability in all groups using both OCT devices, but were relatively higher for SS-OCT than for SD-OCT (low CMT, 0.978 on SS-OCT and 0.954 on SD-OCT, *p* = 0.138; normal CMT, 0.988 on SS-OCT and 0.930 on SD-OCT, *p* < 0.001; high CMT, 0.984 on SS-OCT and 0.948 on SD-OCT, *p* = 0.029; Fig 2, Table 3). A similar tendency was found for the mRNFL thickness measurements, i.e., the degree of repeatability differed between the CMT groups, being highest in the normal CMT group, lower in the high CMT group, and lowest in the low CMT group (low CMT, 0.669 on SS-OCT and 0.639 on SD-OCT, *p* = 0.439; normal CMT, 0.952 on SS-OCT, 0.922 on SD-OCT, *p* = 0.158; high CMT, 0.836 on SS-OCT, 0.803 on SD-OCT, *p* = 0.379; Fig 3, Table 4). The ICCs for GC-IPL thickness measurements showed higher repeatability using SS-OCT than using SD-OCT (low CMT, 0.940 on SS-OCT, 0.911 on SD-OCT, *p* = 0.275; normal CMT, 0.978 on SS-OCT, 0.940 on SD-OCT, *p* = 0.020; high CMT, 0.926 on SS-OCT, 0.899 on SD-OCT, *p* = 0.304; Fig 4, Table 5).

**Table 3:** Repeatability of the two consecutive measurements of macular thickness using the intraclass correlation coefficient and coefficient of variation in the eyes with retinal disease.

		Low CMT group (N = 20)					Normal CMT group (N = 35)					High CMT group (N = 23)				
		SS-OCT		SD-OCT			SS-OCT		SD-OCT			SS-OCT		SD-OCT		
		ICC (95% CI)	CV (%)	ICC (95% CI)	CV (%)	*P*†	ICC (95% CI)	CV (%)	ICC (95% CI)	CV (%)	*P*†	ICC (95% CI)	CV (%)	ICC (95% CI)	CV (%)	*P*†
	**Center**	0.904 (0.431–0.965)	10.5	0.920 (0.844–0.959)	8.9	0.391	0.967 (0.933–0.984)	5.0	0.838 (0.679–0.918)	11.5	0.001	0.969 (0.927–0.987)	5.2	0.745 (0.398–0.892)	20.5	<0.001
**Inner**	**Temporal**	0.956 (0.825–0.989)	5.0	0.972 (0.945–0.986)	4.8	0.251	0.933 (0.864–0.968)	7.0	0.981 (0.962–0.990)	4.2	0.005	0.975 (0.941–0.989)	4.0	0.937 (0.852–0.973)	5.0	0.068
	**Superior**	0.973 (0.889–0.993)	4.8	0.966 (0.933–0.982)	5.0	0.367	0.978 (0.955–0.989)	4.6	0.910 (0.821–0.954)	8.9	0.002	0.977 (0.946–0.990)	4.7	0.946 (0.737–0.935)	5.1	0.085
	**Nasal**	0.956 (0.821–0.989)	5.1	0.786 (0.675–0.892)	19.5	0.007	0.980 (0.959–0.990)	4.4	0.961 (0.843–0.982)	3.9	0.088	0.970 (0.929–0.987)	4.8	0.898 (0.760–0.957)	7.7	0.023
	**Inferior**	0.876 (0.702–0.994)	9.9	0.968 (0.948–0.981)	5.5	0.021	0.992 (0.985–0.996)	1.3	0.971 (0.943–0.985)	4.1	0.005	0.916 (0.802–0.964)	8.7	0.953 (0.889–0.980)	4.2	0.172
**Outer**	**Temporal**	0.983 (0.930–0.996)	4.0	0.927 (0.857–0.963)	7.9	0.015	0.966 (0.930–0.983)	3.9	0.977 (0.955–0.989)	4.7	0.213	0.947 (0.875–0.978)	5.0	0.888 (0.735–0.952)	7.6	0.109
	**Superior**	0.978 (0.910–0.994)	4.1	0.939 (0.880–0.969)	7.7	0.065	0.910 (0.815–0.956)	8.8	0.920 (0.841–0.959)	8.0	0.403	0.952 (0.886–0.980)	4.7	0.943 (0.865–0.976)	5.0	0.390
	**Nasal**	0.960 (0.841–0.990)	5.2	0.966 (0.942–0.980)	5.8	0.405	0.958 (0.914–0.980)	6.6	0.866 (0.734–0.932)	9.1	0.008	0.889 (0.738–0.953)	8.4	0.930 (0.764–0.986)	5.2	0.223
	**Inferior**	0.981 (0.964–0.989)	4.0	0.960 (0.922–0.980)	5.7	0.136	0.990 (0.980–0.995)	1.5	0.994 (0.989–0.997)	1.4	0.153	0.979 (0.950–0.991)	4.4	0.897 (0.758–0.956)	7.5	0.005
	**Average**	0.978 (0.913–0.995)	5.8	0.954 (0.923–0.973)	7.9	0.138	0.988 (0.976–0.994)	4.8	0.930 (0.862–0.965)	6.2	<0.001	0.984 (0.963–0.993)	5.5	0.948 (0.878–0.978)	7.5	0.029

^†^Statistical method of Donner and Zou.

*P* < 0.05 indicates statistically significant difference; A CV of <10% indicates high repeatability

ICC = intraclass correlation coefficient; CI = confidence interval; CV = coefficient of variation; CMT = central macular thickness; SS-OCT = swept-source optical coherence tomography; SD-OCT = spectral domain optical coherence tomography

**Table 4:** Repeatability of the two consecutive measurements of macular retinal nerve fiber layer thickness using the intraclass correlation coefficient and coefficients of variation in the eyes with retinal disease.

		Low CMT group (N = 20)					Normal CMT group (N = 35)					High CMT group (N = 23)				
		SS-OCT		SD-OCT			SS-OCT		SD-OCT			SS-OCT		SD-OCT		
		ICC (95% CI)	CV (%)	ICC (95% CI)	CV (%)	*P*†	ICC (95% CI)	CV (%)	ICC (95% CI)	CV (%)	*P*†	ICC (95% CI)	CV (%)	ICC (95% CI)	CV (%)	*P*†
	**Center**	0.776 (0.100–0.944)	18.6	0.599 (0.349–0.752)	38.3	0.158	0.981 (0.923–0.995)	4.0	0.833 (0.659–0.919)	12.2	<0.001	0.863 (0.677–0.942)	10.0	0.808 (0.683–0.961)	13.2	0.281
**Inner**	**Temporal**	0.965 (0.860–0.991)	3.9	0.536 (0.248–0.714)	40.5	<0.001	0.990 (0.959–0.997)	2.5	0.923 (0.843–0.963)	7.8	<0.001	0.922 (0.817–0.967)	8.8	0.823 (0.583–0.925)	12.0	0.064
	**Superior**	0.482 (0.084–0.871)	40.1	0.472 (0.145–0.674)	45.6	0.485	0.806 (0.587–0.897)	15.7	0.816 (0.708–0.954)	14.6	0.454	0.901 (0.765–0.958)	8.7	0.802 (0.532–0.916)	13.1	0.119
	**Nasal**	0.760 (0.032–0.940)	17.9	0.769 (0.625–0.857)	20.2	0.475	0.980 (0.921–0.995)	3.4	0.972 (0.943–0.986)	3.4	0.248	0.824 (0.584–0.925)	12.6	0.749 (0.409–0.894)	15.5	0.265
	**Inferior**	0.849 (0.390–0.962)	13.2	0.796 (0.670–0.874)	17.9	0.315	0.987 (0.946–0.997)	2.8	0.943 (0.882–0.972)	5.6	0.001	0.862 (0.674–0.941)	9.8	0.901 (0.766–0.958)	8.2	0.288
**Outer**	**Temporal**	0.895 (0.579–0.974)	10.1	0.725 (0.554–0.830)	25.8	0.062	0.956 (0.825–0.989)	5.0	0.906 (0.806–0.954)	8.8	0.058	0.851 (0.648–0.937)	9.4	0.609 (0.477–0.834)	30.1	0.040
	**Superior**	0.925 (0.698–0.981)	6.8	0.574 (0.309–0.737)	39.3	0.002	0.933 (0.730–0.983)	6.9	0.964 (0.927–0.983)	5.4	0.101	0.920 (0.812–0.966)	8.0	0.853 (0.654–0.938)	11.0	0.154
	**Nasal**	0.875 (0.496–0.969)	10.5	0.725 (0.554–0.830)	19.8	0.102	0.968 (0.871–0.992)	4.0	0.944 (0.885–0.973)	6.0	0.126	0.845 (0.635–0.934)	12.3	0.668 (0.218–0.859)	20.1	0.086
	**Inferior**	0.888 (0.551–0.972)	9.8	0.785 (0.652–0.868)	16.8	0.151	0.987 (0.946–0.997)	3.5	0.934 (0.866–0.968)	7.7	0.001	0.937 (0.852–0.973)	9.2	0.735 (0.375–0.888)	16.5	0.007
	**Average**	0.669 (0.228–0.858)	14.5	0.639 (0.477–0.834)	29.4	0.439	0.952 (0.805–0.988)	5.3	0.922 (0.832–0.964)	7.9	0.158	0.836 (0.613–0.930)	9.9	0.803 (0.563–0.905)	15.5	0.379

^†^Statistical method of Donner and Zou.

*P* < 0.05 indicates statistically significant difference; A CV of <10% indicates high repeatability

ICC = intraclass correlation coefficient; CI = confidence interval; CV = coefficient of variation; CMT = central macular thickness; SS-OCT = swept-source optical coherence tomography; SD-OCT = spectral domain optical coherence tomography

**Table 5:** Repeatability of the two consecutive measurements of macular ganglion cell-inner plexiform layer thickness using the intraclass correlation coefficient and coefficient of variation in the eyes with retinal disease.

		Low CMT group (N = 20)					Normal CMT group (N = 35)					High CMT group (N = 23)				
		SS-OCT		SD-OCT			SS-OCT		SD-OCT			SS-OCT		SD-OCT		
		ICC (95% CI)	CV (%)	ICC (95% CI)	CV (%)	*P*†	ICC (95% CI)	CV (%)	ICC (95% CI)	CV (%)	*P*†	ICC (95% CI)	CV (%)	ICC (95% CI)	CV (%)	*P*†
	**Center**	0.838 (0.679–0.918)	14.5	0.820 (0.644–0.959)	16.5	0.433	0.859 (0.431–0.965)	12.2	0.856 (0.714–0.927)	13.9	0.482	0.965 (0.917–0.985)	4.5	0.857 (0.799–0.882)	15.6	0.01
**Inner**	**Temporal**	0.981 (0.962–0.990)	2.1	0.972 (0.945–0.986)	3.8	0.284	0.956 (0.825–0.989)	6.8	0.901 (0.804–0.950)	7.9	0.047	0.945 (0.871–0.977)	6.9	0.883 (0.859–0.893)	9.9	0.107
	**Superior**	0.910 (0.821–0.954)	8.1	0.966 (0.933–0.982)	4.4	0.072	0.973 (0.889–0.993)	8.7	0.854 (0.711–0.926)	10.6	<0.001	0.942 (0.864–0.975)	7.8	0.982 (0.957–0.992)	4.0	0.03
	**Nasal**	0.866 (0.734–0.932)	10.9	0.800 (0.608–0.898)	17.9	0.262	0.956 (0.821–0.989)	6.6	0.766 (0.537–0.882)	21.2	<0.001	0.881 (0.720–0.950)	9.1	0.904 (0.801–0.980)	7.0	0.36
	**Inferior**	0.971 (0.943–0.985)	3.5	0.901 (0.806–0.950)	10.8	0.033	0.976 (0.902–0.994)	8.2	0.889 (0.780–0.944)	9.7	0.001	0.922 (0.816–0.967)	6.5	0.878 (0.849–0.891)	8.9	0.229
**Outer**	**Temporal**	0.977 (0.955–0.989)	3.2	0.927 (0.857–0.963)	9.7	0.043	0.983 (0.930–0.996)	2.1	0.945 (0.890–0.972)	5.6	0.009	0.821 (0.629–0.897)	11.1	0.932 (0.839–0.971)	5.6	0.052
	**Superior**	0.920 (0.841–0.959)	7.1	0.939 (0.880–0.969)	7.7	0.341	0.978 (0.910–0.994)	3.0	0.975 (0.951–0.998)	3.2	0.398	0.918 (0.806–0.965)	8.1	0.899 (0.837–0.952)	9.0	0.365
	**Nasal**	0.867 (0.737–0.932)	10.7	0.774 (0.557–0.885)	20.2	0.198	0.960 (0.841–0.990)	5.4	0.862 (0.727–0.930)	10.4	0.005	0.850(0.813–0.900)	12.2	0.831 (0.737–0.871)	16.8	0.419
	**Inferior**	0.994 (0.989–0.997)	1.0	0.960 (0.922–0.980)	3.0	0.003	0.916 (0.664–0.979)	7.4	0.931 (0.863–0.965)	4.9	0.341	0.869 (0.692–0.945)	10.8	0.865 (0.718–0.985)	13.5	0.48
	**Average**	0.940 (0.872–0.965)	6.8	0.911 (0.864–0.945)	10.4	0.275	0.978 (0.913–0.995)	6.7	0.940 (0.881–0.970)	9.7	0.020	0.926 (0.778–0.960)	8.6	0.899 (0.830–0.931)	10.0	0.304

^†^Statistical method of Donner and Zou.

*P* < 0.05 indicates statistically significant difference; A CV of <10% indicates high repeatability

ICC = intraclass correlation coefficient; CI = confidence interval; CV = coefficient of variation; CMT = central macular thickness; SS-OCT = swept-source optical coherence tomography; SD-OCT = spectral domain optical coherence tomography

## Discussion

In this study, we found that both SS-OCT and SD-OCT had high repeatability for measurement of the thickness of the macula, mRNFL, and GC-IPL in the group with normal eyes. However, the ICCs for SS-OCT were higher than those for SD-OCT overall. In the group with retinal disease, the overall repeatability of thickness measurements was higher for SS-OCT than for SD-OCT; however, the repeatability was lower than that in the group with normal eyes. In the CMT subgroup analysis, SS-OCT showed higher repeatability than SD-OCT regardless of the CMT value, and both OCT devices had relatively low repeatability of mRNFL thickness measurements in eyes with low CMT.

With the recent advent of SS-OCT, researchers have investigated the repeatability of this OCT device in normal eyes. Mansouri et al. investigated the repeatability of all SS-OCT scan protocols in normal eyes and found that repeatability was high for choroidal and retinal thickness measurements when using automated segmentation (ICC = 0.8–0.9).[18] However, they suggested that SS-OCT also had scan artifacts in up to 9% of cases, which should be taken into account when interpreting measurement data. Another study by Lee et al. compared the repeatability of peripapillary RNFL and GC-IPL thickness measurements between SD-OCT and SS-OCT in normal eyes.[19] They reported that the ICCs for peripapillary RNFL thickness measurements were higher on SS-OCT than on SD-OCT, but both OCT devices had ICCs > 0.9 for most clinically relevant subfields. The same trend was found for the GC-IPL thickness measurements, i.e., no clinically relevant difference was detected between two devices. The study by Lee et al obtained macular GC-IPL thickness measurements in 6 subfields,[19] whereas we used the ETDRS map, which is used mainly in retinal research, and there was a similar trend of high repeatability for both OCT devices in normal subjects. Although the study by Lee et al was the first to compare SD-OCT and SS-OCT with regard to measurement of thickness values, the OCT devices used in their study was not from same manufacturer, in that their SS-OCT device was from Topcon and their SD-OCT device was from Carl Zeiss Meditec Inc. (Dublin, CA, USA). This limits the relevance of the findings of their study because use of different segmentation algorithm software may affect repeatability. In contrast, our study compared the repeatability of SD-OCT with that of SS-OCT using devices manufactured by same company (Topcon). We analyzed the OCT images using the same image viewer software, so we were able to evaluate the influence of the laser source more accurately when measuring retinal thickness by excluding segmentation algorithm software factors.

We assessed the repeatability of macular, mRNFL and GC-IPL thickness measurements obtained by SS-OCT in eyes with retinal disease for the first time and divided the retinal disease group into three subgroups according to CMT. The overall ICCs for the retinal disease group were lower than those for the normal group using both OCT devices, with the exception of macular thickness using SD-OCT. We found that the low and high CMT groups had ICCs that were lower than those in the normal CMT group using both OCT devices. This trend was consistent with that seen in previous studies comparing normal eyes with eyes affected by retinal disease using SD-OCT.[20, 21, 29] Lee et al. reported that the ICCs for thickness measurements in groups with normal eyes, eyes with macular edema, and eyes with atrophy were 0.998, 0.985, and 0.903, respectively. Our data also showed high repeatability for macular thickness measurements (ICC > 0.8–0.9 on both OCT devices).

In our study, both OCT devices showed similar repeatability (ICC > 0.8–0.9) for GC-IPL thickness measurements, which is similar to previous findings using SD-OCT.[20] Therefore, both OCT devices had similar intra-device repeatability for measurement of macular and GC-IPL thickness in eyes with retinal disease, regardless of CMT. However, another study performed by Lee et al. suggested that SD-OCT had low repeatability in eyes with ERM, especially in those with high CMT (>450 μm). Shin et al. also reported that repeatability of SD-OCT for GC-IPL measurements was low in eyes with AMD that were out of the normal CMT range.[29] In the present study, retinal diseases were classified by CMT rather than by specific disease because of the small sample sizes. Further studies are warranted to confirm the repeatability of SS-OCT for each specific retinal disease.

Macular RNFL is the thinnest auto-segmentation layer measurable by current OCT devices. We found that both OCT devices showed high repeatability for mRNFL thickness measurements in eyes with retinal disease and normal or high CMT. An SS-OCT study by Min et al. reported that the ICCs for mRNFL thickness measurements were high in eyes with DR with or without macular edema.[30] However, in our study, the repeatability of the mRNFL measurements was significantly lower for both OCT devices in the low CMT group (average ICC 0.669 on SS-OCT vs. 0.639 on SD-OCT). In the low CMT group, SS-OCT had 6 subfields with high ICCs (>0.8), while SD-OCT had no such subfields. This finding may reflect the fact that mRNFL in the low CMT group was far thinner than that in the normal and high CMT groups, which may lead to segmentation error in the detection of the mRNFL. In contrast, high repeatability was observed for the macular and GC-IPL thickness measurements, even in the low CMT group. These differences might reflect differences in the research methods used in the studies. The study by Min et al. used a wide scan protocol (12 × 9 mm), whereas we used a standard-size scan protocol (6 × 6 mm or 7 × 7 mm). In addition, our study included a mixture of retinal diseases, whereas their study included one specific retinal disease. Our study did not investigate the repeatability of peripapillary RNFL thickness measurements, which is important for diagnosis of glaucoma. According to previous reports on the repeatability of peripapillary RNFL thickness measurements obtained by SD-OCT in eyes with retinal disease, the repeatability was relatively higher (with ICCs in the range of 0.8–0.9) than that of our mRNFL measurements. Lee et al. and Shin et al. demonstrated high repeatability when measuring peripapillary RNFL thickness in eyes with ERM and AMD.[21, 29] We attribute this difference to the fact that the peripapillary RNFL is thicker than the mRNFL.

There are several potential explanations for why the ICCs were lower in the retinal disease group than in the normal group in our study. One reason may be the low average visual acuity and another may be an unstable gaze. Auto-segmentation errors because of the change in macular contour are other possible reasons.[31, 32] Overall, SS-OCT showed slightly higher repeatability than SD-OCT for measurements of inner retinal thickness in eyes with retinal diseases in our study. As mentioned above, the SS-OCT system provides faster scan speeds and better image resolution, and uses a longer wavelength light source (1050 nm) than SD-OCT, so can improve the repeatability of inner retinal thickness measurements by reducing segmentation artifacts.[16, 17]

The strength of our study is in the fact that it is the first to compare the repeatability of SS-OCT with that of SD-OCT for measurement of inner retinal thickness in both normal eyes and eyes with retinal disease. Although the measurement of inner retinal layer thickness provided by the OCT device is useful and widely used to evaluate the macular ganglion cell layer and the nerve fiber layer, the reliability of the measurement should be considered when interpreting data from patients with retinal diseases, especially with low CMT. In addition, the scan protocols, image viewer, and OCT devices used in our study were made by same manufacturer, which made it possible to compare only the effects of the laser source, while excluding other confounding factors.

However, there are several limitations to this study. The first is the relatively small sample size. Further prospective studies in larger numbers of patients are needed to confirm our present findings. Second, the retinal diseases included in this study were heterogeneous. In practice, segmentation errors are more likely to be more influenced by specific retinal layer abnormalities rather than CMT. For example, ERM can cause inner retinal segmentation errors, while AMD can cause outer retinal segmentation errors. Finally, the purpose of this study was to compare the repeatability of inner retinal thickness according to the difference in light source between SD-OCT and SS-OCT. However, other than the light source, SD-OCT and SS-OCT also differ in axial resolution (5–6 μm of axial resolution in SD-OCT vs. 8 μm of axial resolution in SS-OCT). This point should be considered in interpreting the results of this study.

Although SS-OCT showed relatively better repeatability for inner retinal thickness measurements in both normal eyes and eyes with retinal disease when compared with SD-OCT, the two OCT devices showed comparable performance. However, the repeatability of mRNFL in the condition of retinal atrophy is low even for SS-OCT. The changes in the retinal layer configuration of the atrophic retina may affect thickness measurements, which should be considered when assessing patients with retinal diseases, especially when combined with glaucoma or neuro-ophthalmologic disorders. A future study comparing the repeatability of the two OCT devices in eyes with glaucoma, in which mRNFL and GC-IPL thickness is clinically significant, would be worthwhile.
